# Seed germination ecology of Alexandra palm (*Archontophoenix alexandrae*) and its implication on invasiveness

**DOI:** 10.1038/s41598-019-40733-0

**Published:** 2019-03-11

**Authors:** Bin Wen

**Affiliations:** Center for Integrative Conservation, Xishuangbanna Tropical Botanical Garden, Chinese Academy of Sciences, Mengla, Yunnan 666303 China

## Abstract

Biological invasions are occurring worldwide, causing enormous economic and ecological damage. Early detection and prediction of invasiveness are the most effective measures to reduce its damage. The Alexandra palm (*Archontophoenix alexandrae*) is a prolific seeder and an alien species widely planted in tropical China. To help understand the invasion risks posed by this species, lab and field experiments on seed germination were conducted. Results show that the seeds only germinate within a temperature range of 20–30 °C and are sensitive to desiccation and high temperature, with seedling inhibition at 35 °C and −0.8 MPa. Complete viability loss was observed after desiccation to water content of 0.17–0.21 g/g or heat treatment for 30 minutes at 60 °C and above. However, appropriate habitats such as the rainforest understory, forest gaps, forest edges, and a rubber plantation are present in Xishuangbanna. Seeds are also frequently consumed by animals; therefore, there is a high potential for Alexandra palm to become an invasive species in Xishuangbanna. Currently, the main barrier to invasion in Xishuangbanna is likely to be the need for seed dispersal into suitable moist, partly shaded, habitats. Understanding the requirements for germination of the Alexandra palm can better inform management strategies for the control of this species.

## Introduction

Every year, a large number of plant species spread across their natural dispersal barriers^1,2^, most often caused by humans both intentionally and accidentally^3,4^. Numerous alien plant species are deliberately planted in artificial habitats outside their native distribution areas, enriching our botanical resources, beautifying our landscapes, and improving our environment, whilst increasing the risk of biological invasion, according to the tens rule^5^, i.e. the number of successful invasive species is positively related to that of introduced. Some of these imported alien plants escape cultivation to establish self-sustaining populations, colonize abandoned lands, enter agricultural fields, or invade natural ecosystems, posing a major threat to biodiversity and human livelihoods globally^6^, resulting in tremendous economic and ecological loss^7,8^. Xu and Qiang documented entrance methods of 259 of China’s exotic invasive plant species, of which all but nine entered China through intentional or unintentional human introduction^3^. For an effective management of these alien plant species, it is essential to establish some simple and effective methods to assess their invasion risk and standardise human species introduction.

Alexandra palm, *Archontophoenix alexandrae*, is a stately and graceful palm with a slender, grey trunk, and large, feather-like leaves, which lends a tropical look to the landscape. It is native to the littoral rainforest of north-eastern Australia, but is now widely planted as an ornamental plant in tropical and subtropical regions of the world^9^. In Xishuangbanna, in tropical southwest China, it is widely cultivated in parks, gardens, roadsides, and residential areas. However, Alexandra palm has the potential to become an invasive species in Xishuangbanna because: (1) it is a prolific seeder, with year-round fruiting and flowering, where a single adult tree produces thousands of viable seeds each year, creating a massive propagule pressure in the surrounding areas^10^; (2) Xishuangbanna has a wet tropical climate, similar to that in its native range, providing a potential base for its invasion owing to climatic similarity^11^; (3) a congener, *Archontophoenix cunninghamiana*, has become an invasive species in several countries, including Australia, Brazil, and New Zealand^12^; and (4) *A. alexandrae* itself is invasive in disturbed moist and wet forests on the island of Hawai’i^9,13^. Thus, it is now rather urgent to carry out work on some key colonization processes probably associated with invasive potential of Alexandra palm in Xishuangbanna. The goal of this study was to address the following questions: (1) What are the barriers preventing *A. alexandrae* from becoming invasive in Xishuangbanna? (2) Which habitat(s) are likely to be most susceptible?

As seed production and germination is the sole mode for Alexandra palm to propagate, knowledge of its seed biology would be very useful for assessing its potential invasiveness. This study conducted both lab and field experiments to investigate the survival and germination of Alexandra palm seeds under diverse conditions.

## Results

### Effects of desiccation on seed water content and viability

Alexandra palm seeds used in this study had a high initial viability (seedling percentage >90%) although their 100-seed weights varied from 49.74 ± 1.13 g to 56.57 ± 0.64 g, and initial water content ranged between 0.54 ± 0.03 g/g to 0.69 ± 0.02 g/g, depending on seed lots.

**Figure 1:**
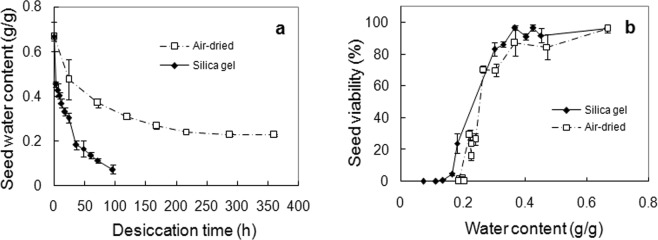
Changes in seed water content during drying treatment (**a**) and effects of drying treatment on seed viability (**b**). Seed water content are means ± SE of eight replicates of single seeds, while seedling values are means ± SE of six replicates of 25 seeds, with survival percentage not shown because not essentially different from seedling percentage.

Silica gel desiccated seeds faster and more thoroughly than air-drying. Those dried by silica gel reached a water content of 0.0726 g/g within 96 h; while air-dried seeds reached equilibrium at *ca*. 0.227 g/g after 288 h, after which water content fluctuated between 0.188 g/g and 0.224 g/g (Fig. 1a; data not shown for air-drying after the 15^th^ day). The seeds were sensitive to desiccation, but not to drying rates. Although seeds dried by silica gel generally retained higher viability than those air-dried at equivalent water content, the difference was limited, as shown by the small gap between viability curves for silica gel- and air-dried seeds (Fig. 1b). Seeds can be desiccated to around 0.3 g/g without essential viability loss (critical water content), demonstrating that seeds are only moderately sensitive to desiccation. Complete loss of viability occurred at around 0.18 g/g, with a WC_50_ (the water content corresponding to 50% seedling loss) of around 0.25 g/g under both drying regimes.

### High-temperature tolerance of quiescent seeds

**Figure 2:**
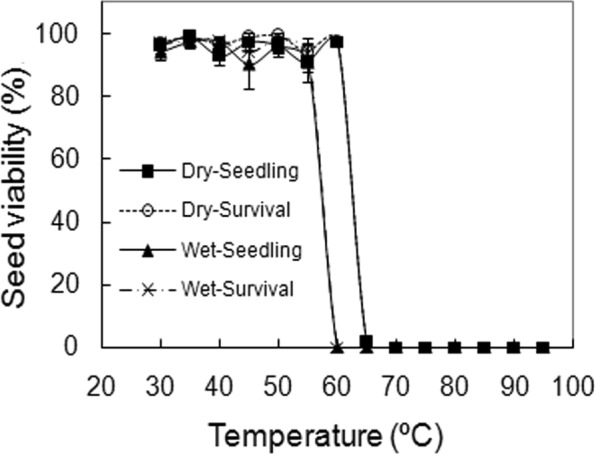
Effects of 30-min heating treatment at a constant temperature between 30 °C and 95 °C on viability of surface-dried (dry) or submerged (wet) seeds. Survival and seedling values are means ± SE of six replicates of 25 seeds.

The seeds were found to be highly sensitive to extreme high temperatures, and both temperature and seed hydration significantly affected seed viability (Table 1; *p* < 0.001 for all factors assessed by survival, but only for temperature when assessed by seedling). For both surface-dried and submerged seeds, 30 min at temperatures ≤55 °C did not reduce viability, but no seeds were viable at 65 °C and above. Viability curves for surface-dried and submerged seeds largely overlapped, however, at 60 °C all dried seeds survived but no submerged seeds did (Fig. 2).

**Table 1:** Summary of the generalized linear models to test the variables affecting seedling and germination or survival of Alexanndra Palm seeds.

Experiment	Effect	df	Seedling			Germination/Survival		
			Estimate ± SE	*Z*-value	*P*-value	Estimate ± SE	*Z*-value	*P*-value
Incubation temperature	Temperature	7	0.175 ± 0.026	6.711	<0.001	0.222 ± 0.027	8.348	<0.001
High-temperature tolerance	Temperature	13	−1.231 ± 0.158	−7.772	<0.001	−14.565 ± 1.842	−7.906	<0.001
	Hydration status	1	−0.796 ± 0.701	−1.136	0.256	−49.781 ± 6.857	−7.260	<0.001
	T × H	13	−0.098 ± 0.104	−0.949	0.343	6.367 ± 0.926	6.874	<0.001
Continuous heat treatment	Heating duration	9	−0.777 ± 0.038	−20.59	<0.001	−0.777 ± 0.038	−20.59	<0.001
Periodic heat treatment	Heating duration	8	−0.614 ± 0.037	−16.760	<0.001	−0.620 ± 0.038	−16.400	<0.001
Water availability	Water potential	9	−2.812 ± 0.216	−13.040	<0.001	−1.369 ± 0.0773	−17.710	<0.001

### Effect of incubation temperature on seed germination

**Figure 3:**
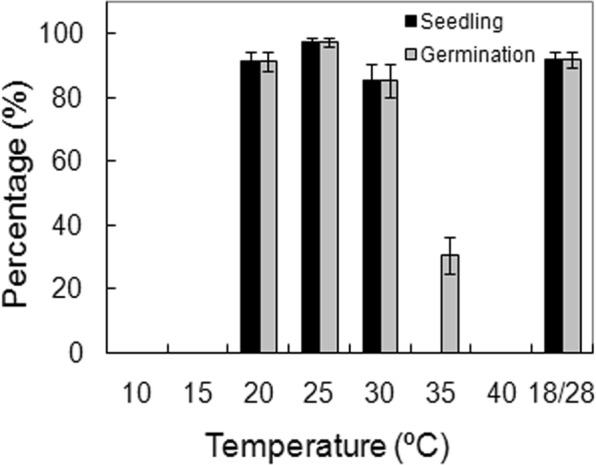
Effects of incubation temperatures on seed germination. Seeds were incubated at a constant temperature between 10 °C and 40 °C, and alternating temperatures of 18/28 °C. Germination and seedling values are means ± SE of six replicates of 25 seeds.

Temperature was a key factor affecting germination (*p* < 0.001 for both germination and seedling, Table 1). High germination occurred only at constant temperatures between 20 °C and 30 °C and in alternating temperatures of 18 °C/28 °C; maximum germination occurred at 25 °C. Despite the tropical origin of this species, its germination is sensitive to high-temperatures: around 30% of seeds incubated at 35 °C germinated, but they failed to form morphologically normal seedlings (Fig. 3). No germination occurred at 40 °C and above or at 15 °C and below, but more than half of the seeds incubated at 10 °C and 15 °C germinated after being moved to 25 °C three months later (data not shown).

### Effects of water availability on seed germination

**Figure 4:**
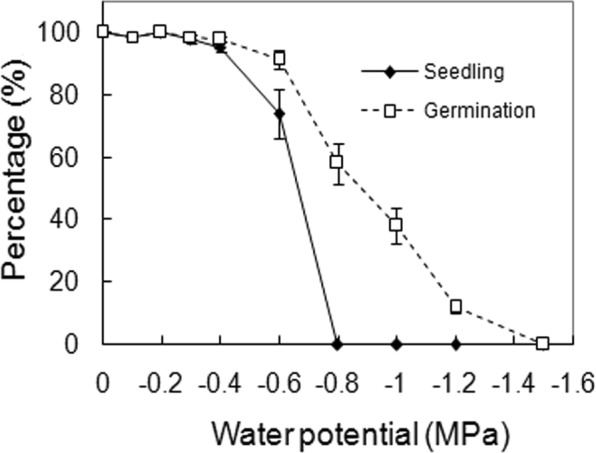
Effects of water potentials on seed germination. Seeds were incubated at 25 °C, and water potentials were created by PEG 8000. Germination and seedling values are means ± SE of six replicates of 25 seeds.

Water availability also had a significant effect on both germination and seedling percentage (*p* < 0.001 for both, Table 1). Seedling percentage was obviously lower than germination percentage when water stress exceeded −0.4 MPa, with seedling and germination being completely inhibited at −0.8 and −1.5 MPa, respectively (Fig. 4). However, most ungerminated seeds germinated after released from the stress (data not shown).

### Effects of continuous heat treatment on seed viability

**Figure 5:**
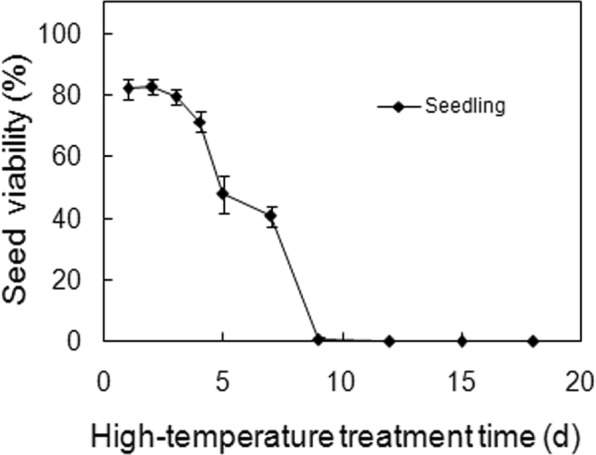
Effects of continuous heating treatment at 40 °C on seed viability. Seeds were subjected to heat shock at 40 °C for indicated periods of time, and then moved to incubation at 30 °C. Seedling values are means ± SE of six replicates of 25 seeds, with survival percentage not shown because not essentially different from seedling percentage.

The seeds were sensitive to continuous treatment at 40 °C (*p* < 0.001 for both survival and seedling, Table 1). Up to three days made little difference, four days reduced seed viability, five days halved seed viability, and there were no surviving seeds after ten days (Fig. 5).

### Effects of periodic high temperature on seed germination

**Figure 6:**
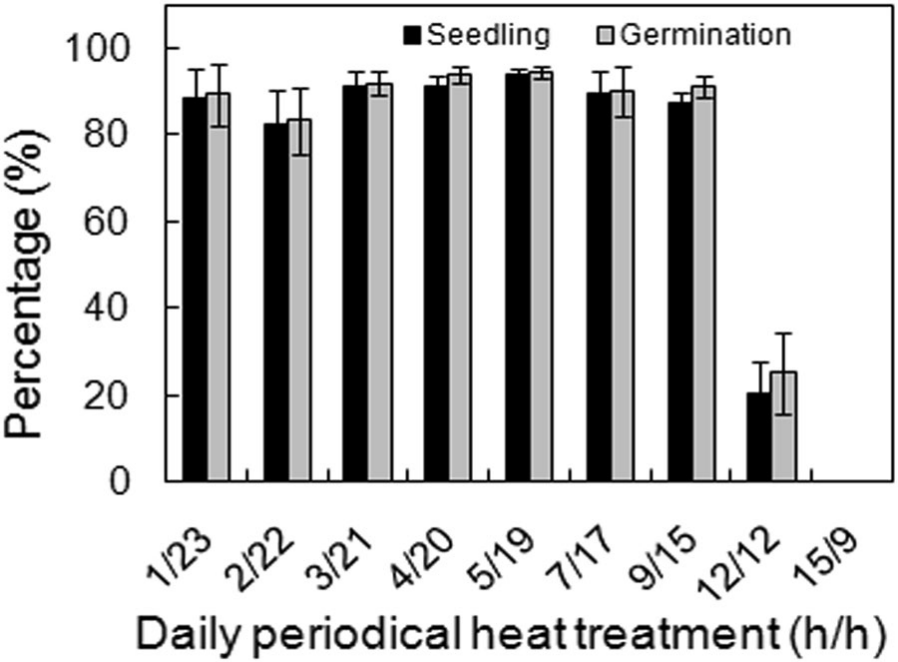
Effects of daily periodic heating treatment on seed germination. Seeds were subjected to 40 °C and 30 °C (h/h) alternately. Germination and seedling values are means ± SE of six replicates of 25 seeds.

Daily periodic high temperatures significantly affected seed germination (*p* < 0.001 for both germination and seedling, Table 1). Most seeds germinated and formed normal seedlings when exposed to a high temperature of 40 °C for up to 9 h, but 12 h of exposure depressed seedling and germination percentage to about 25%, and seeds did not germinate after 15 h exposure (Fig. 6).

### Field seed viability and germination

**Figure 7:**
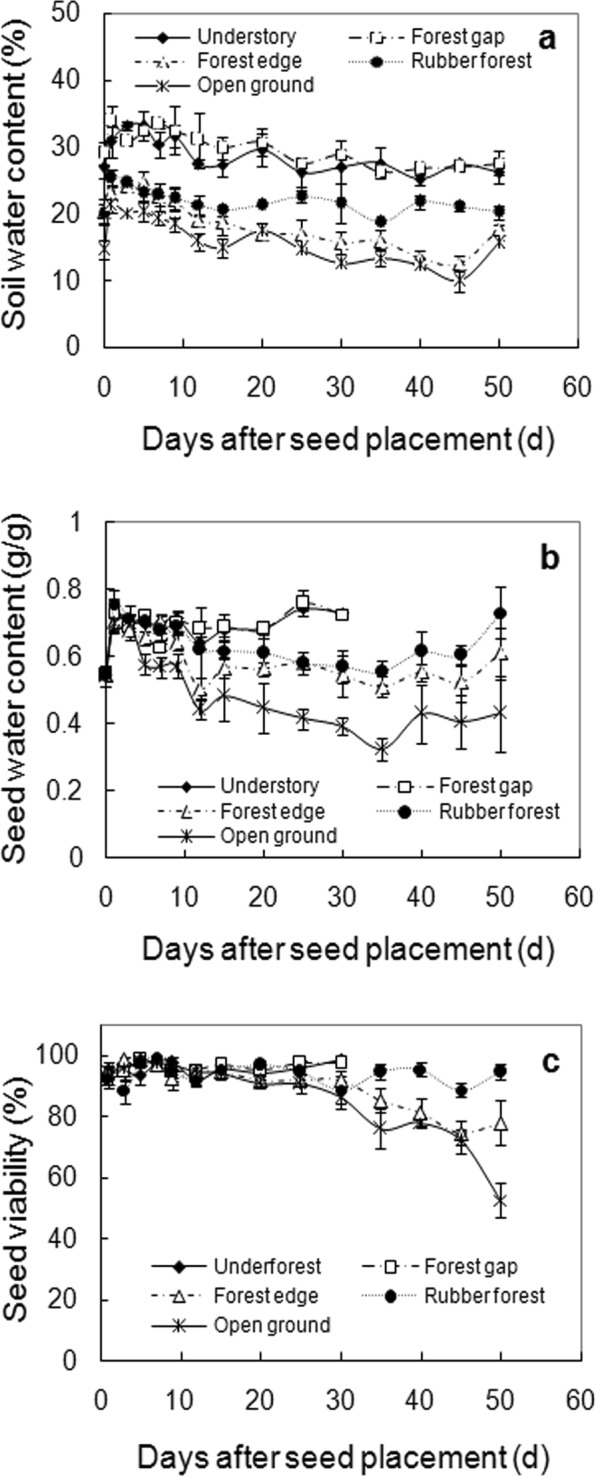
Changes in soil water content (**a**), seed water contents (**b**) and seed viability (**c**) in the five habitats. Soil water contents are means ± SE of three replicates, seed water contents are means ± SE of eight replicates of single seed, and seedling values are means ± SE of six replicates of 25 seeds, with survival percentage not shown because not essentially different from seedling.

Soil water content in the field varied depending on the weather and differed between habitats. Soil samples from the understory (Plot A) and forest gap (Plot B) always had the highest water content, with an average of 28.63 ± 2.61% and 29.79 ± 2.51%, respectively, followed by those from the rubber plantation (Plot D; 21.79 ± 1.75%) and the forest edge (Plot C; 18.87 ± 3.82%), with the driest soil in open ground (Plot E; 15.97 ± 3.34%, Fig. 7a).

Water content of seeds placed in these habitats varied accordingly. Rainfall caused moisture to increase in the beginning, from 0.54 g/g to 0.69–0.75 g/g. After which, seeds in open ground progressively lost water, except during the final period, where the lowest water content of 0.32 g/g was documented, this is close to the critical water content (0.3 g/g). A slight moisture decline was also detected in seeds placed in rubber forest and forest edge up to the 35^th^ day, while those placed in the understory and forest gap retained high water content, no less than 0.63 g/g. During the experiment, seed moisture values below the critical water content (0.3 g/g) were not detected and only seeds placed in open ground dried below the initial water content (0.54 g/g, Fig. 7b).

Similar changes were found in seed viability. From the 30^th^ day after being placed in open ground, seeds showed a progressive decline in viability, with viability halved by the 50^th^ day. A slight but significant viability loss also occurred in seeds placed at the forest edge, which retained 75% viability at the final stage. Seeds in the understory and forest gap had no viability loss, with germination >95% until the 30^th^ day after placement. Although a fluctuation in viability was detected for seeds sampled from the rubber plantation, there was a high occurrence of germination at the final stage (Fig. 7c).

Seeds began to germinate in the field from the 12^th^ day, and around two thirds of seeds placed in the understory and forest gap had germinated by the 30^th^ day. After this, most seeds were consumed so the experiment discontinued at these two sites. Seeds placed in forest edge and rubber plantation germinated later and slower, half had germinated by the 50^th^ day. The seeds placed in open ground did not germinate. One year later, many seedlings remained in the rubber plantation (data not shown).

The seeds that had been consumed in the understory and forest gap plots were replaced in mid-December 2016 and covered with metal mesh to exclude animals. Neither water nor viability loss were detected in this seed cohort, but unlike the November cohort, none of the seeds germinated, possibly because temperatures were low in the field then (data not shown).

## Discussion

The present study investigated the seed germination requirements of Alexandra palm, seed responses to high temperature and water stress, and seed fate in diverse habitats. Seeds germination only occurred within a narrow temperature range (20–30 °C), which is similar to the soil surface temperature regime in tropical rainforest in Xishuangbanna^17,18^. Germination and seedling percentage were seriously inhibited when water potential fell below −0.6 MPa. This sensitivity to temperature and water stress probably explained why no seeds germinated when placed on bare ground. In these aspects, Alexandra palm has a lot in common with a local rainforest tree, *Baccaurea ramiflora*; however, the seeds of *B. ramiflora* lose moisture faster on bare ground^14,16^. This contrasted with alien invasive species in the same area such as, bamboo piper^19^, Mexican sunflower^20^, spiny amaranth^21^, and three invasive Asteraceae weeds^22^.

Previous reports^23,24^ have shown that palm seeds are recalcitrant, i.e. sensitive to abiotic stress, but only moderately recalcitrant^25^ according to our findings. Seeds can be desiccated to 0.3 g/g by silica gel without essential viability loss, and seeds which suffered both low temperatures (10 °C and 15 °C) and water stress (≤−0.8 MPa) germinated after being released from stresses. These attributes make the seeds capable of retaining viability for a relatively long time in the field and allowing for germination in diverse habitats. Even on bare ground, although none germinated, half the seeds were viable for 50 days.

Alexandra palm has been widely cultivated in tropical and subtropical China for decades^26^. Although it has not yet become naturalized or invasive, there are now very large human-sustained populations. Previous studies found that invasive plants had seed traits in common which may contribute to plant invasiveness, such as small seed mass and high seed output^27,28^. Like most invasive plant species, Alexandra palm produces a large number of viable seeds every year, however, these are recalcitrant, while most invasive plants produce orthodox seeds, i.e. seeds tolerant to abiotic stress. However, exceptions exist such as *Ardisia crenata* and congeneric *Archontophoenix cunninghamiana*, which often invade shaded habitats such as mesic forests^12^. This study also found that shaded and mesic habitats are suitable for Alexandra palm, only that nowadays almost all trees are planted in open areas, where the recalcitrant seeds are prone to lose viability after shedding. Hence, seed recalcitrance of Alexandra palm can be considered as a barrier to invasion, and dispersal into the susceptible habitats may therefore be the limiting step. However, these are not insurmountable, as the diaspore is eaten intentionally^29,30^. The small (for palms, *ca*., mean diameter 10.687 ± 0.048 mm) red fruits are very attractive to frugivorous birds. In two 15-minute sessions, several red-whiskered bulbuls (*Pycnonotus jocosus*, the commonest frugivore in parks, gardens, and roadsides across tropical China) and two sooty-headed bulbuls (*P. aurigaster*) were found swallowing whole fruits, and a squirrel (*Callosciurus inornatus*) scraping off the flesh and dropping the seeds from an Alexandra palm tree in XTBG (Corlett, R.T., 2018. Personal communication). These predators may help to disperse seeds into forests^31,32^.

After being introduced to a new region, to enter the suitable habitats is a critical step for successful naturalization or invasion of an alien species. According to the schema questions of van Kleunen *et al*.^33^, Alexandra palm has been picked up and introduced, meanwhile the appropriate environment is present there; but there are difficulties in its approach to the appropriate sites. So, the current barrier is to reach the susceptible habitats though it is unknown whether there are any more on the way to invasion. Differing from Zhou *et al*. who attributed failure of rubber trees (*Hevea brasiliensis*) to expand into natural forests in Xishuangbanna to its poor seed dispersal^34^, this study emphasized the importance of seed sensitivity to desiccation and high temperature. To my knowledge, the present study is the first report to relate seed storage behaviour to plant invasiveness. Alexandra palm seeds have abundant predators which can be potential dispersers, and these seeds can germinate in shaded habitats which are susceptible to invasion. This study suggested that Alexandra palm is a potential invader in Xishuangbanna and that if seeds are dispersed to shaded habitats the invasion process^35^ of this palm may progress. At present the key measures for management and invasion prevention of this species is to restrict its human introduction into any forests, both artificial and natural.

Early detection and prediction have been a major aim of ecological research since biological invasions have come into focus^36–38^, because they allow maximization of any control effort. Biological invasion is a complex process composed of several transitions, e.g. transportation, release and establishment^35^. At present, Alexandra palm has a large human-sustained population, but not a self-sustained one in Xishuangbanna. Instead of taking invasion as a whole, this study focused on the likelihood for Alexandra palm to enter the next stage along the invasion process. Lab experiments were designed to investigate its germination requirements and seed tolerance to stresses, meanwhile field experiments carried out to test its fitness to local habitats. Findings show that there is an abundance of habitats suitable for its growth in Xishuangbanna, which clarified its possibility as an invader and the barrier at present. This is vital information that can be utilised for management and control of this species.

## Materials and Methods

### Seed materials

Alexandra palm (*Archontophoenix alexandrae*) seeds used in this study were collected from trees planted in a residential quarter in Menglun Town, where Xishuangbanna Tropical Botanical Garden, Chinese Academy of Sciences (21°55′N, 101°15′E) is located. As fruits on the same inflorescences usually have very uniform maturity, with most of them shedding within around two weeks, the entire inflorescences were harvested when mature fruits were shedding, from June to December of 2016. There was a total of five seed lots used throughout the entire study, composed of more than 20 inflorescences from over 15 trees, with seeds used in each experiment coming from the same seed lots. After harvest, epicarps were manually removed by friction in a mesh bag under running water (hereafter fruits with epicarps removed are referred to as seeds) and surface dried at ambient conditions for one day. Then, 100-seed weights (10 replicates of 100 seeds), initial water content (dry weight basis, 8 replicates of single seed, dried at 103 °C for 17 h), and viability (using germination test of 25 seeds × 6 replicates, see below) were determined. The remaining seeds were stored in plastic bags at 15 °C for no more than one week before all experiments were set.

### Laboratory experiments

As high temperature and desiccation are common stresses to seeds in the tropics, the following experiments were designed to investigate their effects on seed viability and germination.

#### Seed desiccation tolerance

Two methods were used to assess seed desiccation tolerance: rapid drying and slow drying. In rapid drying, seeds were buried in silica gel desiccant, which was renewed when its colour changed; while in slow drying, seeds were air-dried in a monolayer under ambient conditions (*ca*. 17–22.5 °C and RH 70–90%, averages 19.6 °C and RH 81.7% during the experimental period, recorded at 5-min intervals using a data logger, model RC-4HC, Elitech Electric Company Limited, Jiangsu, China). In both methods, seeds were sampled regularly for water content and viability assessment, at 3–24 h interval for rapid drying, and 1–5 d interval for slow drying.

Correlated seed water content and viability data from this experiment was subjected to Probit analysis to determine critical seed water content (water content corresponding to 15% viability loss) and WC_50_ (water content corresponding to 50% viability loss), performed with SPSS v.16.0 (SPSS Inc., Chicago, Illinois, USA).

#### High temperature tolerance

A temperature gradient ranging from 30 °C to 95 °C with steps of 5 °C was established using a water bath to investigate the effect of extreme high temperatures on seed viability^14^. Two large triangular flasks containing 150 seeds in a monolayer each were used for each testing temperature. The seeds in one flask remained surface-dried during heating while those in the other flask were submerged in deionized water at the same temperature as the water bath. After heating for half an hour at the indicated experimental temperature, the seeds were removed from the water bath and immediately sown for viability assessment.

#### Effect of incubation temperature on seed germination

Germination was conducted by sowing seeds on 1% water agar in Petri dishes, and randomly assigned to incubators set at a constant temperature regime between 10 °C and 40 °C at 5 °C increments and an alternating temperature regime of 18/28 °C (in total, 8 temperature regimes were tested), with a 12 h photoperiod of 25 μ mol m^−2^ s^−1^ irradiance provided by white fluorescent lamps.

#### Effects of water availability on seed germination

Polyethylene glycol (PEG) 8000 was used to create solutions with water potentials of 0, −0.1, −0.2 to −1.5 MPa^15^. Seeds sown on defatted cotton moistened with testing solutions in Petri dishes were incubated at 25 °C in light for germination. To minimize moisture loss during the experiment, the Petri dishes were sealed in resealable double-clear plastic bags. Seed germination was scored twice a week, with defatted cotton and testing solutions refreshed simultaneously. Eight weeks later, the ungerminated seeds were transferred to defatted cotton moistened by deionized water to test if they were still viable.

#### Effects of continuous high-temperature treatment on seed viability

Seeds were sown on water agar as previously described, and placed in an incubator set at 40 °C. The heat stress treatment periods ranged from 1 to 18 d, a total 10 treatments. After heating for a given period, dishes containing seeds were moved to an incubator set at 30 °C to check seed viability.

#### Effects of periodic high temperature on seed germination

In order to investigate the effects of varying high-temperature length on seed germination, seeds sown on 1% agar were artificially transferred between 40 °C and 30 °C incubators every day so that they were exposed to daily heat shock for the duration of 1 to 15 h.

### Field experiments

#### Plot establishment

In Xishuangbanna Tropical Botanical Garden (XTBG) there is a remnant of wet seasonal tropical rainforest (XTBG rainforest), consisting of an 80 ha area, located in a valley with both its slopes, at an altitude of *ca*. 530–580 m. Since the establishment of the Botanical Garden in 1959, this piece of rainforest has been protected for conservation and scientific study^16^. Trees reach heights of 35–45 m and there are three inconspicuous tree layers. Important tree species include *Tetrameles nudiflora, Pometia tomentosa, Terminalia myriocarpa, Antiaris toxicaria, Garcinia cowa* and *Baccaurea ramiflora*.

Although Alexandra palm has been planted as an ornamental plant in artificial habitats outside its range, it grows naturally in Australian rainforests, so five distinct habitats around the XTBG rainforest were chosen to carry out field experiments as follows:

Plot A: under the XTBG rainforest, in a valley, at 560 m altitude, with a diverse plant cover, dense canopy and clean understory;

Plot B: in a small gap in the XTBG rainforest, on a slope, at 575 m, with some *Musa acuminata* plants;

Plot C: at the edge of the XTBG rainforest, at 570 m, with diverse herbs;

Plot D: under a rubber (*Hevea brasiliensis*) monoculture near the XTBG rainforest, at 585 m, with a continuous canopy and clear understory;

Plot E: on relatively bare ground near the XTBG rainforest, at 580 m, with direct sunlight on sunny days. There are sparse *Anthocephalus chinensis* trees growing there.

#### Field seed experiments

The experiments began in early November 2016. To investigate seed viability changes in the field, one 1 m × 1 m quadrat was set up in each plot. After removing covering herbs and litter, approximately 3,500 seeds were placed in a monolayer on the ground. Once every 1–5 d (with shorter interval early), they were sampled in a 25 seeds × 6 replicates style for viability assessment and 8 seeds for water content until the 50th day. At the same time, soil samples were taken from each plot to monitor changes in soil moisture.

### Seed Viability and Germination Assessment

For germination and viability tests in this study, 25 seeds × 6 replicates were used for each treatment. Unless stated otherwise, the seeds were sown on 1% agar, incubated at 25 °C, and scored once a week for at least eight weeks. When a visible radicle could be discerned, the seed was considered to have germinated in germination requirement experiments (germination) or survived in stress treatment experiments (survival); the seed was removed and considered to have formed seedling when a shoot grew (seedling). Before experiments were completed, ungerminated seeds were subjected to a cut test to assess viability; white, firm embryos were viable, and brown, soft embryos were nonviable.

### Data analysis

In this study, seedling and germination or survival percentages were separately taken as dependent variables for data analysis. As germination data usually lack a normal distribution, a generalized linear model (glm) was used to analyse seedling and germination or survival with a binomial error distribution and logit-link function. All applied environmental factors were treated as fixed effects, interactions between factors also evaluated. Except for the desiccation tolerance experiment, all analyses were done with the statistical programming language R (v.3.3.3) (R Foundation for statistical Computing, Vienna, Austria).

## Data Availability

All data was presented within this paper.
